# Analysis of Dermal Papilla Cell Interactome Using STRING Database to Profile the *ex Vivo* Hair Growth Inhibition Effect of a Vinca Alkaloid Drug, Colchicine

**DOI:** 10.3390/ijms16023579

**Published:** 2015-02-05

**Authors:** Ching-Wu Hsia, Ming-Yi Ho, Hao-Ai Shui, Chong-Bin Tsai, Min-Jen Tseng

**Affiliations:** 1 Institute of Molecular Biology and Department of Life Science, National Chung Cheng University, Chia-yi 621, Taiwan; E-Mail: welovearthurhsia@gmail.com; 2 Institute of Stem Cell and Translational Cancer Research, Chang Gung Memorial Hospital, Taoyuan 333, Taiwan; E-Mail: myho@cgmh.org.tw; 3 Graduate Institute of Medical Sciences, National Defense Medical Center, Taipei 114, Taiwan; E-Mail: haoai@ndmctsgh.edu.tw; 4 Department of Ophthalmology, Chia-yi Christian Hospital, Chia-yi 600, Taiwan

**Keywords:** alopecia, colchicine, dermal papilla, interactome, Protein ANalysis THrough Evolutionary Relationships, Search Tool for the Retrieval of Interacting Genes/Proteins

## Abstract

Dermal papillae (DPs) control the formation of hair shafts. In clinical settings, colchicine (CLC) induces patients’ hair shedding. Compared to the control, the *ex vivo* hair fiber elongation of organ cultured vibrissa hair follicles (HFs) declined significantly after seven days of CLC treatment. The cultured DP cells (DPCs) were used as the experimental model to study the influence of CLC on the protein dynamics of DPs. CLC could alter the morphology and down-regulate the expression of alkaline phosphatase (ALP), the marker of DPC activity, and induce IκBα phosphorylation of DPCs. The proteomic results showed that CLC modulated the expression patterns (fold > 2) of 24 identified proteins, seven down-regulated and 17 up-regulated. Most of these proteins were presumably associated with protein turnover, metabolism, structure and signal transduction. Protein-protein interactions (PPI) among these proteins, established by Search Tool for the Retrieval of Interacting Genes/Proteins (STRING) database, revealed that they participate in protein metabolic process, translation, and energy production. Furthermore, ubiquitin C (UbC) was predicted to be the controlling hub, suggesting the involvement of ubiquitin-proteasome system in modulating the pathogenic effect of CLC on DPC.

## 1. Introduction

Hair loss (alopecia, effluvium) is one of the most unwanted side effects of medications. Chemotherapy-induced alopecia (CIA), often found in cancer patients, is one of the most adverse drug reactions (ADR) of chemotherapy and affects the body image to bring severe psychological devastation to cancer patients and compromises their quality of life [1]. Two different modalities of drug-induced alopecia have been reported: anagen effluvium and telogen effluvium [2]. Anagen effluvium is resulted from the apoptosis of rapidly dividing matrix epithelial cells of hair bulbs of anagen HFs and it occurs within days to weeks of drug administration. Telogen effluvium is due to the drug induced premature rest of anagen HFs, which can be observed two to four months after starting the therapy.

HFs are mini organs and have become increasingly valuable as an experimental model of developmental biology [3]. The similarity in the HF development of humans and rodents makes the HFs of rodents a good model to study the disease progress of HFs with the administration of drugs [4]. The hair cycle has three major phases: anagen, catagen and telogen [5]. Anagen is the growth phase of HFs and DPs direct hair bulb matrix epithelial cells to produce hair shafts. The cell number and size of the DP determine the size of the HF and thickness of hair fibers [6]. Catagen is a degenerative phase with the apoptosis of HF epithelial cells. The shrinkage of HFs leads to the release of DPs from hair bulbs [7]. In telogen, scant cytoplasmic DPs are quiescent in a poor extracellular matrix environment in the dermis [5]. DPs are specialized mesenchymal cells in hair bulbs and function as the control center of HFs [3]. They regulate the phase transition in the hair cycle with the cycling change of alkaline phosphatase (ALP) activity in DPs. The level of ALP activity is a well-established DP marker [8]. DPs and *in vitro* cultivated DPCs can induce the neogenesis of HFs in animal models [9,10]. Therefore, DP of the HF functions both a chemical and physical niche for the progeny of keratinocyte stem cells in the follicular epithelium that regenerate the cycling portion of the HF and generate the hair shaft [11]. DP cell numbers fluctuate over the hair cycle, and hair loss is associated with gradual depletion/atrophy of DP cells. More recent works showed that the size of this niche is dynamic and actively regulated and reduction in DP cell number per follicle is sufficient to cause hair thinning and loss. When DP cell number falls below a critical threshold, HFs with a normal keratinocyte compartment fail to generate new hairs [12,13].

Colchicine (CLC), a microtubule-depolymerizing agent (MDA) and antineoplastic drug, disrupts the assembly of actins and microtubules and causes metaphase arrest in cells [14,15]. CLC blocks the HF activity and induces hair loss* in vivo* [16,17]. Harms indicated that CLC therapy induced patients’ alopecia [18]. The toxicity of CLC also induced patient effluvium [19,20]. However, the influence of CLC on DPs is as yet unknown. 

Interactome (the network of interacting proteins) consists of the regulatory pathways to determine the cellular behavior. An advantage of PPI maps is that researchers can gain understanding about specific signaling pathways and the functions of individual proteins [21].

In our previous report, we demonstrated that CLC not only inhibited the proliferation of DPCs, but also stimulated DPCs to release histone H4 to retro-modulate themselves [22]. Due to the niche effect of DP on HF [11], we further examined the effect of CLC on HFs *ex vivo* and DPCs *in vitro*. Using a combined approach of 2-DE and nano-LC-ESI-MS/MS analysis, the protein patterns of DPCs in the presence and absence of CLC were profiled. The differentially expressed proteins were further analyzed using STRING database to derive a genome-scale PPI network and the potential signaling pathways, which may reveal the pharmacological mechanism of CLC on DPCs.

## 2. Results and Discussion

### 2.1. The Effect of Colchicine (CLC) on Hair Growth

**Figure 1:**
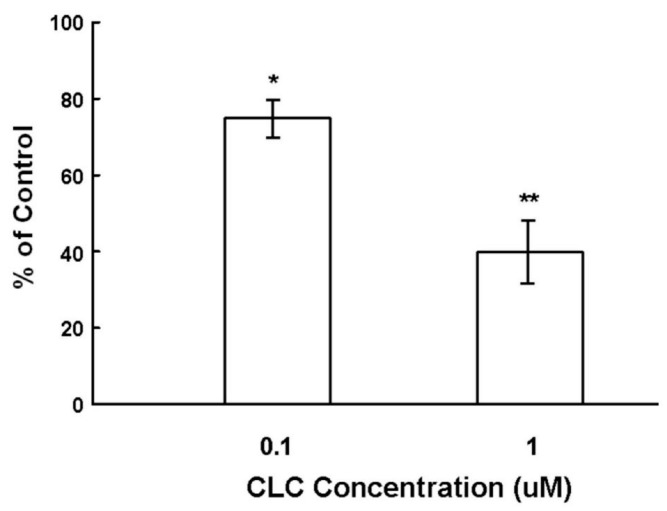
Colchicine (CLC) inhibited the *ex vivo* elongation of hair fiber. The organ cultured rat vibrissa hair follicles (HFs) were treated with CLC (0.1 and 1 µM) for seven days and the length of HF fiber was determined. The vibrissa HFs with DMSO treatment was taken as control (100%). Data were expressed as the mean from three batch triplicate experiments;* bars*, ±SD. * *p* < 0.05, ** *p* < 0.01 *vs.* control group.

To examine the effect of CLC on the hair shaft elongation, organ cultured Sprague-Dawley (SD) rat vibrissa HFs were exposed to CLC of different concentrations (0.1 and 1 µM) for seven days. As shown in Figure 1, CLC significantly inhibited the hair shaft elongation in a dose-dependent manner from 12.5% ± 2.3% by 0.1 µM to 58% ± 4.5% by 1 µM after seven days.

### 2.2. CLC Induced Morphological Changes of DPCs

**Figure 2:**
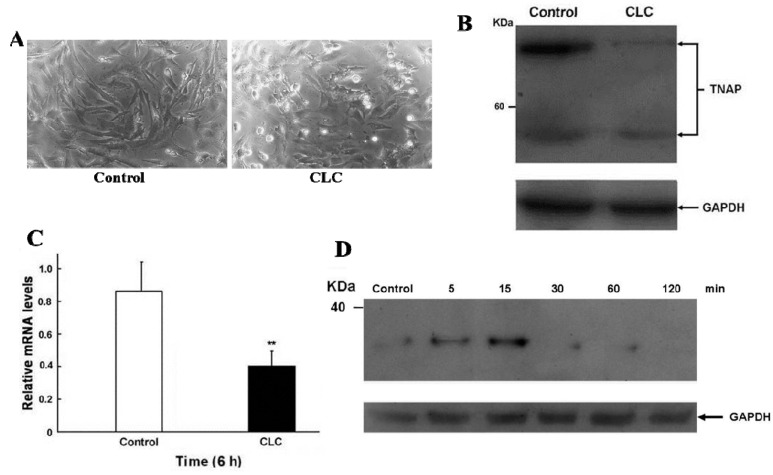
CLC induced morphological changes (**A**), reduced ALP expression (**B**,**C**) and increased IκBα phosphorylation (**D**) of dermal papillae cells (DPCs)* in vitro*. DPCs were treated with DMSO (control) or 2 µM CLC (CLC) for 6 h except for panel D. (**A**) The morphological alteration of DPCs. Magnification: 200×; (**B**) The protein level of alkaline phosphatase (ALP) in DPCs was detected by Western blot (WB). Arrows indicate the positions of tissue nonspecific ALP (TNAP). In all WB analyses, the loading and transfer of equal amounts of total lysate (20 µg per lane) were confirmed by immuno-detection of Glyceraldehyde 3-phosphate dehydrogenase (GAPDH); (**C**) The expression level of ALP mRNA in DPCs was analyzed by qRT-PCR. The level of ALP mRNA was normalized to that of GAPDH. Data were expressed as the mean from triplicate experiments;* bars*, ±SD, ** *p* < 0.01 *vs*. control group; (**D**) the time course of IκBα phosphorylation of DPCs after CLC treatment at indicated times was analyzed by WB.

After exposing DPCs to 2 µM CLC for 6 h, the morphologies of DPCs were altered significantly, from elongated fibroblast-like to discoid or polygonal epithelial-like cells (Figure 2A) without obvious DPC apoptosis as determined by trypan blue exclusion. Interfere with normal cytoskeletal dynamics by the addition of CLC brings great stresses on cells and causes the destabilization of polymerized microtubule and the loss of cell shape [23]. Accordingly, 2 µM CLC disrupted the microtubules of DPCs to cause the morphological changes of DPC even within 6 h. Our previous data have shown that the long-term (72 and 120 h) proliferation of DPCs was inhibited under the same treatment [22].

### 2.3. CLC Down-Regulated the Expression of ALP in DPCs

Since ALP activity is the widely used cell marker of DPCs [8], the ALP level of DPCs in the presence or absence of 2 µM CLC was determined by Western blot (WB) and real-time quantitative reverse transcription-PCR (qRT-PCR) analysis. CLC (2 µM) significantly down-regulated the expression of ALP in both protein and transcript levels of DPCs after 6 h (Figure 2B,C)

#### The Implication of ALP Down-Regulation on the Biological Function of DPCs

DPs, which mainly consist of DPCs, play a key role in directing matrix epithelial cells to produce hair shafts in anagen [3,11]. CLC has been shown to induce animal and patients alopecia [17,18,19,20]. The ALP expression of DPCs is recognized both as the marker of DPC activity and an index for the degree of drug-induced damage to DPCs within HF dystrophy [10]. Cultured DPCs with strong ALP activity interact with epithelial cells to induce the neogenesis of HFs *in vivo* [9]. As shown in Figure 1, CLC inhibited the *ex vivo* elongation of rat vibrissa hair fiber in a dose-dependent manner. Although compared to non-proliferative DPCs, the active proliferating matrix cells are more sensitive to CLC, we deduce that the impact of CLC to the activity (ALP) of DPCs also participated in the inhibition of hair shaft formation resulted from CLC. Collectively, the CLC-treated DPCs with reduced ALP activity were suggested to be inactivated and have lost the ability to direct epithelial cells to produce hair shafts and new HFs.

### 2.4. CLC Induced IκBα Phosphorylation of DPCs

The time course of protein levels of phosphor-IκBα of DPC were monitored by WB following 2 µM CLC treatment (Figure 2D). The phosphorylation levels of IκBα were increased in 5 min and the increment was about 3.5-fold at 15 min. Unto 30 min of treatment; levels of phosphor-IκBα were reduced to near undetectable level. The phosphorylation of IκBα promoted its ubiquitination and degradation in proteosome that led to release of NF-κB from the inhibitory IκBα-NF-κB complex. The released NF-κB then translocated into nucleus to regulate downstream genes [24].

MDAs reorganized microtubules to activate NF-κB and induce NF-κB-dependent gene expression [25,26]. Cytoskeleton disruption by CLC induced a rapid NF-κB activation in human neuroblastoma IMR-32 cells [27]. An acute NF-κB activation was also found when challenged human DPCs with TNF-α and IL1-β [28]. We deduce that the IκBα/NF-κB signaling pathway may participate in the dysfunction of DPCs exposed to CLC.

### 2.5. Two-Dimensional (DE) Analysis for the Effect of CLC on DPCs

**Figure 3:**
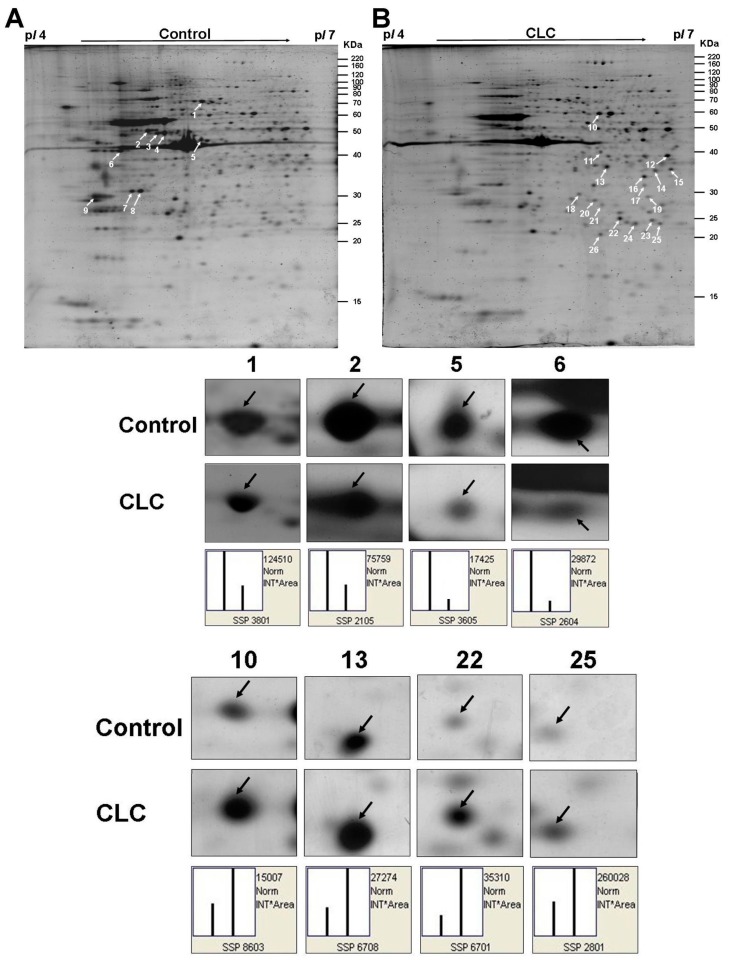
Computed image analysis of Sypro-ruby stained 2-DE gels. (**A**) The proteomic signature of DPCs; (**B**) The proteomic signature of DPCs treated with 2 µM CLC for 6 h. The magnified images of four down-regulated and four up-regulated protein spots were also shown below the 2-DE gels, respectively. White arrows indicated the positions of identified proteins on 2-DE gels. The standard spot (SSP) number was the number attributed to each protein spot by PDQuest software. Each SSP number uniquely identified that protein.

The representative 2-DE maps of DPCs treated with or without 2 µM CLC for 6 h were depicted and each gel resolved up to ~654 protein spots (Figure 3A,B). The image analysis indicated that 26 protein spots changed their quantities greater than twofold after treating DPCs with CLC. Those protein spots were excised, in-gel trypsin digested and analyzed by the nano-LC-ESI-MS/MS to obtain the MS/MS peak lists. Twenty-six protein spots, nine down-regulated and 17 up-regulated, were identified to match to candidate proteins using Mascot online search engine (Table 1 and Table 2). Both horizontal adjacent down-regulated protein spots 3 and 4 were identified to be protein disulfide-isomerase A6 (Pdia6). The other two horizontal adjacent down-regulated protein spots 7 and 8 were also both identified to be Annexin A5 (Anxa5). Therefore, seven proteins were proteomic identified to be down-regulated after treating DPCs with 2 µM CLC for 6 h. The pattern of protein changes of CLC-treated DPC, except Psmb7, is quite different from those of taxol-treated DPCs [29].

#### 2.5.1. The Seven Proteins Down-Regulated in DPCs by CLC

Table 1 lists the identities of the seven down-regulated proteins in DPCs treated by 2 µM CLC for 6 h. Among seven down-regulated proteins, heat shock cognate 71 kDa protein (Hspa8, Hsc70) belongs to the heat shock protein 70 family. Hsc70 is a constitutively expressed molecular chaperone and 85% identity with heat-shock protein 70. Hsc70 functions as an ATPase and facilitates correct folding of nascent polypeptides [30]. The reduction of Hsc70 was associated with induction of apoptosis and normal aging in primate retina [31]. Furthermore, Hsc70 is highly associated with vimentin (Vim), one of the down-regulated proteins, and suggested to participate in stabilizing the intermediate filaments [32]. Vimentin, a major constituent of the intermediate filament family of proteins, is ubiquitously expressed in normal mesenchymal cells and is known to maintain cellular integrity and provide resistance against stress. The expression of Vim is controlled by cell configuration, and down-regulation of Vim causes the impaired migration and adhesion of cancer cells [33,34].

**Table 1:** Identities of the down-regulated proteins in DPCs treated by 2 µM CLC for 6 h.

Spot	Protein Name ^a^	Gene Name ^b^	Accession No. ^c^	MOWSE Score ^d^	Theoretical *Mr* (Da)/pI ^e^	% Coverage	Representative Peptides ^f^
**Stress**							
1	Heat shock cognate 71 kDa protein	*Hspa8*	P63018	2594	71,055/5.37	69.7	K.DAGTIAGLNVLR.I; R.ARFEELNADLFR.G; R.IINEPTAAAIAYGLDKK.V
**Energy**							
2	ATP synthase subunit, mitochondrial	*Atp5b*	P10719	1680	56,318/5.19	70.9	K.VLDSGAPIKIPVGPETLGR.I; R.AIAELGIYPAVDPLDSTSR.I; R.IPSAVGYQPTLATDMGTMQER.I
**Protein turnover**							
3, 4	Protein disulfide-isomerase A6	*Pdia6*	Q63081	1498	48,542/5.00	56.6	R.GSTAPVGGGSFPNITPR.E; K.IFQKGESPVDYDGGR.T; K.HQSLGGQYGVQGFPTIK.I
5	Eukaryotic initiation factor 4A-II	*Eif4a2*	Q5RKI1	996	46,601/5.33	45.2	K.MFVLDEADEMLSR.G; K.QFYINVEREEWK; R.GIDVQQVSLVINYDLPTNR.E.
**Structure**							
6	Vimentin	*Vim*	P31000	888	53,757/5.06	39.1	R.LGDLYEEEMR.E; R.KVESLQEEIAFLK.K; R.ISLPLPNFSSLNLR.E
**Signal transduction**							
7, 8	Annexin A5	*Anxa*5	P14668	1601	35,779/4.93	79.0	R.ETSGNLENLLLAVVK.S; K.WGTDEEKFITILGTR.S; K.QAYEEEYGSNLEDDVVGDTSGYYQR.M
9	14-3-3 protein ε	*Ywhae*	P62260	1067	29,326/4.63	65.5	R.YLAEFATGNDR.K; K.LICCDILDVLDK.H; K.LICCDILDVLDKHLIPAANTGESK.V

^a^ Protein name: name of each matched protein listed in SwissProt.2011 database; ^b^ Gene name: name of each matched protein listed in SwissProt.2011 database; ^c^ Accession no.: SwissProt.2011 database accession number; ^d^ MOWSE score: the score of identified matched protein in Mascot on-line search engine; ^e^ Theoretical *Mr* (Da)/pI: theoretical molecular weight and pI value of the identified matched; ^f^ M: oxidized methionine.

ATP synthase subunit β, mitochondria 1 (Atp5b) is a subunit of mitochondrial ATP synthase. A decreased expression of Atp5b has been documented in malignant tumors when compared with its level in normal tissues [35,36]. Mitochondria aggregate as clusters and move on the cytoskeleton. CLC depolymerizes microtubules to disrupt mitochondria chains, which are required for mitochondria to respond to the alteration in local energy demands [37]. Protein disulfide-isomerase A6 (Pdia6) is a chaperone protein that inhibits aggregation of misfolded proteins. The ER resident Pdia6 is an attenuator of the unfolded protein response; it interacts covalently with activated IRE1 and facilitates the decay of active IRE1 [38]. Eif4a2 (eukaryotic initiation factor 4A2), a protein-synthesis initiation factor, belongs to the DEAD box helicase family, functions to resolve RNA secondary structures and participates in the binding of mRNA to the 40S ribosome during the initiation phase of eukaryotic translation [39]. The Eif4a2 mRNA synthesis and translation was found to associate preferentially with the growth-arrested (quiescent) state [40].

The 14-3-3 protein ε (Ywhae), a member of highly conserved 14-3-3 family proteins, binds to phosphoserine-containing proteins to mediate signal transduction, and heterodimerizes with 14-3-3 protein ξ (Ywhaz). The down-regulation of 14-3-3 proteins (β, σ, ξ) was found during the transition of anagen to catagen [41]. A proteomic study showed that Ywhae is critical for mediating the PPI in the mitogen-activated protein kinases (MAPK) signal module for modulating NF-κB translocation/activity [42]. Down-regulation of Ywhae and annexin A5 was observed in lung adenocarcinoma treated with tobacco-specific nitrosamine 4-(Methylnitrosamino)-1-(3-pyridyl)-1-butanone [43]. Annexin A5 (anxA5) is a phospholipid binding protein that efficiently binds to phosphatidylserine and it was down-regulated in an epithelial cell treated with rifaximin [44].

#### 2.5.2. The Seventeen Proteins Up-Regulated in DPCs by CLC

The expression of 17 proteins in DPCs was up-regulated by CLC (Table 2). Peroxiredoxin 6 (*Prdx6*), one of the highly conserved peroxidase family proteins, exhibits anti-oxidant character and prevents cell from peroxide-induced cytotoxicity. It has been shown that NF-κB acts as a stress-sensing molecule that determines optimal regulation of Prdx6 transcription for fine tuning the delicate redox balance necessary for the maintenance of cellular functions [45]. Protein disulfide-isomerase A3 (*Pdia3*) gene encodes a protein in the endoplasmic reticulum that interacts with lectin chaperones calreticulin and calnexin to modulate the folding of glycoproteins that are newly synthesized. The protein PDIA3 has been found to be active in several other locations and reactions, for example, interactions in the nucleus which involve DNA repair, DNA damage recognition, and apoptosis [46].

**Table 2:** Identities of the up-regulated proteins in DPCs treated by 2 µM CLC for 6 h.

Spot	Protein Name ^a^	Gene Name ^b^	Accession No. ^c^		MOWSE Score ^d^		Theoretical *Mr* (Da)/pI ^e^	% Coverage	Representative Peptides ^f^
**Stress**									
22	Peroxiredoxin-6	*Prdx6*	O35244		1145		24,860/5.64	76.8	R.DLAILLGMLDPAEK.D; K.LIALSIDSVEDHFAWSK.D; R.VVDSLQLTASNPVATPVDWK.K
**Protein turnover**									
10	Protein disulfide-isomerase A3	*Pdia3*	P11598		1679		57,044/5.88	62.4	K.VDCTANTNTCNK.Y; R.ELNDFISYLQR.E; K.VVVAESFDDIVNAEDK.D
11	26S proteasome non-ATPase regulatory subunit 13	*Psmd13*	B0BN93		1214		43,075/5.55	64.4	K.ITVNKVELLVMK.A; K.SAWGQQPDLAANEAQLLR.K ; K.ETIEDVEEMLNNLPGVTSVHSR.F
13	60S acidic ribosomal protein P0	*Rplp0*	P19945		706		34,365/5.19	49.2	K.EDLTEIRDMLLANK.V; R.VLALSVETDYTFPLAEK.V; K.AFLADPSAFAAAAPVAAATTAAPAAAAAPAK.V
19	Proteasome subunit β type-7	*Psmb7*	Q9JHW0		435		30,250/8.13	33.2	K.FRPDMEEEEAK.K; K.LDFLRPYSVPNK.K; K.LPYVTMGSGSLAAMAVFEDK.F
23	Proteasome subunit α type-2	*Psma2*	P17220		571		26,024/6.92	59.4	R.GYSFSLTTFSPSGK.L; K.HIGLVYSGMGPDYR.V; K.LVQIEYALAAVAGGAPSVGIK.A
26	Protein DJ-1	*Park7*	O88767		505		20,190/6.32	63.5	K.GAEEMETVIPVDIMR.R; K.GAEEMETVIPVDIMR.R; K.TQGPYDVVVLPGGNLGAQNLSESALVK.E
**Structure**									
15	PDZ and LIM domain protein 1	*Pdlim1*	P52944		101		36,018/6.79	11.9	K.VAASVGNAQK.L; R.SAMPFTASPAPGTR.V; R.LVGGKDFEQPLAISR.V
18	F-actin-capping protein subunit β	*Capzb*	Q5XI32		813		30,952/5.69	44.5	R.RLPPQQIEK.N; R.STLNEIYFGK.T; R.KLEVEANNAFDQYR.D
25	Transgelin	*Tagln*	P31232		414		22,645/8.87	46.3	K.AAEDYGVTK.T; K.KYDEELEER.L; R.EFTDSQLQEGK.H
**Signal transduction**									
12	Annexin A1	*Anxa1*	P07150		229		39,147/69.7	12.7	K.DITSDTSGDFR.N; K.TPAQFDADELR.A; K.GVDEATIIDILTK.R
16	Annexin A3	*Anxa3*	P14669		1347		36,569/5.96	56.8	K.ALLTLADGGRDESLK.V; K.NLRDDISSETSGDFR.K; R.GMGTDEDTLIEILTTR.T
**Metabolism**									
14	Malate dehydrogenase, cytoplasmic	*Mdh1*	O88989	655		36,631/6.16		36.5	K.DLDVAVLVGSMPR.R; K.FVEGLPINDFSR.E; K.VIVVGNPANTNCLTASK.S
17	3-mercapto-pyruvate sulfur-transferase	*Mpst*	P97532	525		33,205/5.88		33.3	R.AQPEHVISQGR.G; R.HIPGAAFFDIDR.C; K.THEDILENLDAR.R
20	Isoamyl acetate-hydrolyzing esterase 1	*Iah1*	Q711G3	649		28,386/5.63		55.4	R.SVDIPKER.V; R.DCGTDVLDLWTLMQK.D; K.GAGLENPVAVTIFFGANDSTLKDENPK.Q
21	Latexin	*Lxn*	Q64361	430		25,735/5.77		34.1	K.QVTVSCTAEVLYPR.M; K.VQTVQQASKEDIPGR.G; R.AASVAENCINYQQGTPNK.V
24	Glutathione *S*-transferase P	*Gstp1*	P04906	510		23,652/6.89		52.9	K.YEELQQTAGR.H; R.TEAESWYQTK.Y; R.TTAENEFVMLK.K; K.ALPGHLKPFETLLSQNQGGK.A

^a^ Protein name: name of each matched protein listed in SwissProt.2011 database; ^b^ Gene name: name of each matched protein listed in SwissProt.2011 database; ^c^ Accession no.: SwissProt.2011 database accession number; ^d^ MOWSE score: the score of identified matched protein in Mascot on-line search engine; ^e^ Theoretical *Mr* (Da)/pI: theoretical molecular weight and pI value of the identified matched; ^f^ M: oxidized methionine.

The 26S proteasome non-ATPase regulatory subunit 13 (Psmd13) is a regulatory subunit of the 26S proteasome, which involved in the ATP-dependent degradation of ubiquitinated proteins. Recent report showed that *Psmd13* gene silencing suppressed the production of proinflammatory mediators by modulating ubiquitin-proteasome system-mediated neuro-inflammation *via* the down-regulation of IκBα degradation and NF-κB activation in LPS-stimulated BV2 microglia [47]. Proteasome subunit β type-7 (Psmb7) is also a component of the proteasome, a macromolecular machine integral to cellular proteolytic degradation capability [48]. Proteasome subunit α type-2 (*Psma2*) gene encodes a member of the peptidase T1A family that is a 20S core α subunit strongly interacts with other proteasomes. It may have a potential regulatory effect on other components of the proteasome complex through tyrosine phosphorylation [48].

The 60S acidic ribosomal protein P0 (Rplp0) is a component of the 60S subunit of ribosomes and ribosomes catalyze protein synthesis. Proteomic study showed that Rplp0 was up-regulated in Jurkat cells during heat stress-induced apoptosis [49]. Protein DJ-1 (Park7), a peptidase, acts as a redox-sensitive chaperone and plays an important role in attenuating oxidant stress and promoting cell survival [50]. Through binding and inhibiting a deubiquitination enzyme, Cezanne, Park7 enhanced NF-κB nuclear translocation and cell survival [51]. PDZ and LIM domain protein 1 (Pdlim1, Clim1), a cytoskeletal protein, involves in the assembly and maintenance of stress fibers [52].

Latexin (Lxn) is an endogenous carboxypeptidase inhibitor (CPI), inhibitor for metallo-carboxypeptidases (MCPs) and a negative regulator of hematopoietic stem cells (HSCs) [53]. It has been reported that DPCs exhibited HSC activity and repopulated the mouse hematopoietic system [54]. Latexin inhibits the self-renewal of HSCs by facilitating the lodgment of HSCs within a bone marrow niche to maintain HSC homeostasis [55]. There were two upstream NF-κB regulatory elements involved in the control of cell type-specific expression of rat latexin gene [56]. Transgelin (TAGLN) is an actin cross-linking/polymerization protein that belongs to the family of actin-associated proteins, and involved in the migration of epithelial cells by interacting with actin or promoting podosome formation [57]. Transgelin in the kidney was up-regulated in repopulating mesangial cells *in vivo* and supported their migratory and proliferative repair response after injury [58].

Cytoplasmic malate dehydrogenase (Mdh1) is important in transporting NADH equivalents across the mitochondrial membrane, controlling tricarboxylic acid (TCA) cycle pool size [59]. A proteomic analysis indicated that the activation of the gluconeogenesis caused up-regulation of Mdh and annexin A1 in thick ascending limb of Henle’s loop (TALH cells) in response to osmotic stress [60]. Annexin A1 (Anxa1) has been shown to promote metastasis through constitutively activate NF-κB by interaction with the IκB kinase (IKK) complex in breast cancer cells [61].

Mpst (3-mercaptopyruvate-sulfurtransferase), an antioxidant enzyme, is one of the three enzymes involved in synthesizing H_2_S, which is increased under inflammatory conditions or sepsis in mammalian tissues. Knockout mice study indicated a positive interlink between Anxa1 and H_2_S pathway in the control of inflammation [62]. Glutathione *S*-transferases (GSTs) play an important role in the protection of cells against xenobiotics and lipid hydroperoxides generated by oxidative stress. Glutathione *S*-transferase P1 (Gstp1) prevented LPS-induced excessive production of pro-inflammatory factors through inhibiting LPS-induced MAPK including ERK, JNK and p38 as well as NF-κB activation in RAW264.7 macrophage-like cells [63].

Recent proteomic study showed that human keratinocytes treated with glucose oxidase (source of oxidative stress) could up-regulate expression of several proteins involving in stress response, including Pdia3 and Protein DJ-1, and in cytoskeletal organization, including Annexin A2 and F-actin-capping protein subunit β (Capzb) [64]. F-actin-capping proteins bind in a calcium-independent manner to the fast growing ends of actin filaments (barbed end), thereby playing a role in the regulation of cell morphology and cytoskeletal organization [65]. Nevertheless, the detailed molecular mechanisms of many identified proteins affected by CLC in DPCs require further investigation.

### 2.6. Major Identified Proteins Associated with Protein Turnover and Metabolism

**Figure 4:**
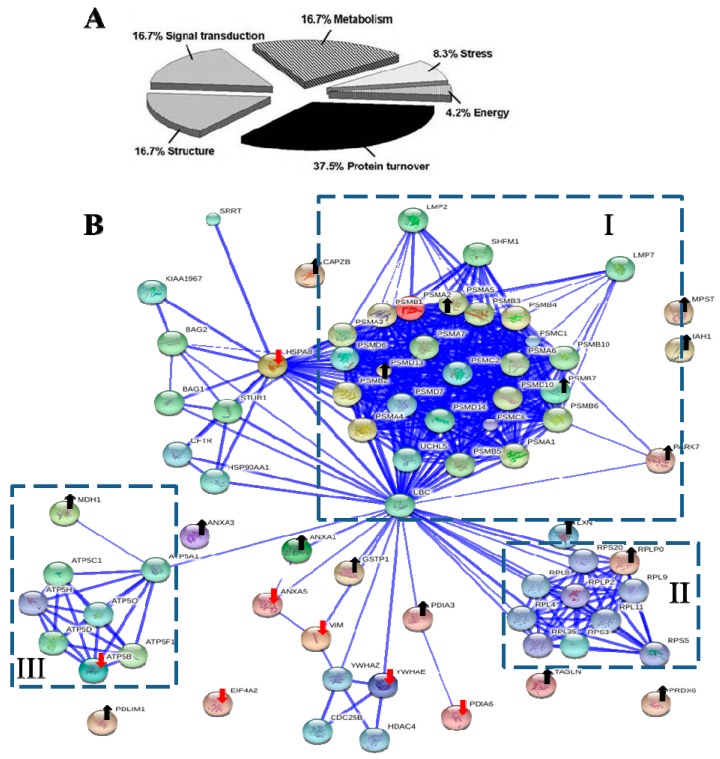
The biological functional distribution and PPI network analysis. (**A**) The pie chart of 24 differentially expressed proteins. The percentage of the proteins in each group was also described; (**B**) The PPI network of 24 differentially expressed proteins. The dot-lined square indicated a functional protein cluster. Downward red and upward black arrows indicate down-regulated and up-regulated proteins, respectively (see Table 1 and Table 2 for description). The PPI were shown in the confidence view produced by STRING database. Thicker blue lines represented the stronger associations.

Base on Protein ANalysis THrough Evolutionary Relationships (PANTHER) classification system [66], the functional distribution of seven down-regulated and 17 up-regulated proteins were each classified into five groups (Table 1 and Table 2), respectively. The major functions of these proteins were associated with protein turnover (37.5%), metabolism (16.7%), structure (16.7%), and signal transduction (16.7%) (Figure 4A).

### 2.7. The Interactomic Analysis Derived a PPI Network with 3 Protein Clusters

Interactomics holds great promise in understanding the molecular mechanism of cells affected by medications. To examine the clusters of 24 differentially expressed proteins and their associated partners and their PPIs, the STRING database [67] was used to deduce enriched protein clusters and generated a PPI network (Figure 4B). The computed predictions indicated that the PPI map contained 1 large (I), and 2 small (II, III) protein clusters.

The symmetric connected cluster I contains proteins of multicatalytic proteasome complex, which participates in protein metabolic process, including proteasome subunit α type-2 (Psma2) and other α-type subunits, proteasome subunit β type-7 (Psmb7) and other β-type subunits, as well as 26S proteasome non-ATPase regulatory subunits 10, 13 and 14 (Psmd10, Psmd13 and Psmd14). The α-type and β-type subunit complexes are the subunits of 20S core of proteasome, whereas Psmd10, Psmd13 and Psmd14 belong to the 19S regulator [48]. Ubiquitin-labeled proteins are degraded viathe ubiquitin-proteasome pathway, which controls the distribution, stability and function of cellular proteins [48,68]. The MDA vincristine enhanced proteasome-mediated degradation and disappearance of tubulin in neural cells, which is inhibited by a proteasome inhibitor MG132 [69].

Cluster II and III are 2 small protein clusters. Cluster II is a symmetric connected translation-related protein cluster, including 60S acidic ribosomal protein P0 (Rplp0), Rplp2, Rpl4, Rpl8, Rpl9, Rpl11, Rps3, Rps5, Rps20, and Rpl35. Cluster III were ATPase networks primarily involved in energy production, such as subunits of mitochondrial ATP synthase (F-ATPases, F1FO-ATPase) Atp5a1, Atp5b, Atp5c1, Atp5d, Atp5f1, Atp5h, Atp5o and Mdh1.

#### Ubiquitin C (UbC) was Predicted as a Controlling Hub Protein

Controlling hubs are the principal proteins with a great number of interactions in a PPI network. We search STRING database for predicting controlling hubs against 24 identified proteins as seeds. The ubiquitin C (UbC) was predicted to be the computed major controlling hub; this protein acts as the connector of three clusters (Figure 4B). *Ubc* is a protein-coding gene and encodes a polyubiquitin precursor. Free ubiquitin is created from UbC by deubiquitinases [70]. Conjugation of ubiquitin monomers or polymers can lead to various effects within a cell, depending on the residues to which ubiquitin is conjugated. Ubiquitination has been associated with protein degradation, DNA repair, cell cycle regulation, kinase modification, endocytosis, and regulation of other cell signaling pathways [71,72]. The ubiquitin system has been known to play diverse roles of in NF-κB activation [24]. Therefore, the network analysis reveals potential roles of ubiquitin-proteasome system in the pathogenic effect of CLC on PDCs.

## 3. Experimental Section 

### 3.1. Preparation of CLC Solution and Primary Culture of SD Rat Vibrissa DPCs

CLC was dissolved in DMSO to make a stock solution of 100 mM, and then stored in dark at 4 °C in aliquots. CLC and DMSO were purchased from Sigma (St. Louis, MO, USA). Primary culture of SD rat vibrissa DPCs was prepared as previous described [22,29].

### 3.2. Organ Culture of SD Rat Vibrissa HF for Hair Fiber Elongation Analysis

Fresh intact vibrissa HFs of SD rats were isolated with a jewelry-maker forceps [73]. Isolated cleaned vibrissa HFs, which were first sterilized with an antibiotic wash medium (RPMI-1640 with 20% FCS, 2 mM l-glutamine, 50 IU/mL penicillin, and 50 µg/mL streptomycin, 10 µg/mL tetracycline, 150 µg/mL chloramphenicol, 10 µg/mL gentamycin sulfate, 4% fungizone) for 30 min in a 5% CO_2_ humidified atmosphere incubator at 37 °C. Sterilized HFs were maintained in William’s E medium (containing 50 IU/mL penicillin, and 50 µg/mL streptomycin) in individual wells of 24-well plates for a 7-day culture period at 37 °C in a 5% CO_2_ humidified atmosphere incubator. The HF organ culture assay was used to evaluate the effect of CLC on the elongation of HF fiber. CLC was added to the HF culture on the first day at final concentrations of 0.1 and 1 µM, respectively. Each HF shaft length was determined under a binocular dissecting microscope, fitted with an eyepiece measuring graticule and measured after 7 days. The length of the HF fiber was defined as the distance between its base and cut tip of the fiber. Cell culture reagents were purchased from Hyclone (Logan, UT, USA). Eight-week-old SD rats were obtained from National laboratory animal center (Taipei, Taiwan).

### 3.3. WB and qRT-PCR Analysis

The protein concentration of DPC lysate was determined using the Bradford protein assay reagent. Samples (20 µg) were resolved in a 15% SDS-PAGE and proteins were electro-transferred onto an Immobilon-P membrane. The protein levels of ALP, phospho-IκBα and GADPH were detected by WB with anti-TNAP, anti-phospho-IκBα, and anti-GADPH, respectively, following the procedure described previously [22]. Immunoblots were scanned and quantified by ImageJ. Trizol reagent was used to isolate total DPC RNA. The qRT-PCR was performed to measure the levels of ALP and GAPDH mRNAs according to the previous described protocol [22].

### 3.4. Two-DE and Image Analysis

DPCs were treated or untreated with 2 µM CLC for 6 h. Trypan blue staining indicated the viability of DPC is greater than 95%. DPCs were washed with cold wash buffer (10 mM Tris-HCl, pH 8.0) and solubilized by sonication in lysis buffer (7 M urea, 2 M thiourea, 2% CHAPS, bromophenol blue, 2% DTT and 0.5% IPG buffer). A whole cell lysate (250 µg) was applied onto an IPG strip (130 × 3 × 0.5 mm, pH 4–7) which was rehydrated for 16 h at 20 °C. The IEF was performed on an Ettan IPGphor II (GE Healthcare, Pittsburgh, PA, USA) with 200 V for 1 h (200 V-h), 500 V for 1 h (500 V-h), 1000 V for 1 h (1000 V-h), a gradient of 8000 V (4000 V-h) and a “step-n-hold” of 8000 V for 6.75 h. After IEF, IPG strips were equilibrated with the equilibration buffer (6 M urea, 2% SDS, 0.05 M Tris-HCl, pH 8.8, 20% glycerol and 0.5% (*w*/*v*) DTT) for 15 min and blocked in the equilibrating buffer by substituting DTT with 4.5% (*w*/*v*) iodoacetamide for 15 min. Each focused IPG strip was separated by a 12.5% SDS-PAGE under a constant voltage of 300 V till finish. Sypro Ruby-stained 2-DE gels were scanned with Typhoon 8600 system (GE Healthcare, Pittsburgh, PA, USA) and analyzed using PDQuest 2-DE image analysis software (Bio-Rad, Hercules, CA, USA). To reduce variations among 2-DE gels resulted from the procedure of staining and destaining, a triplicate 2-DE analysis per sample was performed. Manual matching was done when necessary. 2-DE reagents were purchased from GE Healthcare (Pittsburgh, PA, USA).

### 3.5. In-Gel Protein Digestion and Protein Identification by Nano-LC-ESI-MS/MS

Protein spots of interest were recovered from gels and dried in vacuum. In-gel tryptic digestion was performed and the resulting peptide mixture was extracted in 50% ACN containing 0.1% (*w*/*v*) TFA. Desalted extracted trypsin digested peptides were separated and analyzed by nano-LC-ESI-MS/MS as previous described [22]. The identification of protein was based on MS/MS peak lists, which were generated by the Mascot Distiller program (v2.3.2, Matrix Science, London, UK) using default parameters with Mascot online search engine (v2.2.0.6, Matrix Science, London, UK) against SwissProt 2011 database (524420 sequences; *Rattus norvegicus* taxonomy). All the searching parameters have been described previously [22].

### 3.6. PANTHER and STRING Database Analysis

Accession numbers of identified proteins were analyzed for their biological process and interactions using PANTHER classification system (available online: http://www.pantherdb.org) and STRING database version 9.1 (available online: http://string.embl.de) [67], respectively.

### 3.7. Statistical Data Analysis

Data were processed in a model of Student’s paired *t*-tests to evaluate the significance of differences using SPSS analytical software. The value of *p* for each data is * *p* < 0.05, ** *p* < 0.01 *vs*. control group.

## 4. Conclusions

The present study demonstrates the CLC-modulated DPC interactome provided by STRING database and suggests following conclusions. First, a CLC-induced multi-factor pathogenesis on DPCs is derived from this proteomic data. Second, a sub-lethal dose of CLC reduced the ALP expression of DPC to cause inactivation of DPCs. Third, proteins with functions of protein turnover, metabolism, structure, and signal transduction are greatly affected by CLC. Forth, ubiquitin C (UbC) is predicted by STRING database as a major controlling hub, implying the involvement of ubiquitin-proteasome system in mediating CLC effect on DPCs. However, the detailed molecular mechanism of the pathogenic effect of CLC on DPCs will be further investigated. Finally, the interactomic research can be applied to discover proteins in DPCs related to drug-induced effluvium.
